# Assessing the Impact of the Portfolio Workshop and the Use of the Rubric Matrix for Reflective Writing on Medical Faculty and Students

**DOI:** 10.7759/cureus.59530

**Published:** 2024-05-02

**Authors:** Puja Singh, Sudhir Saxena, Smriti T Pandey, Puja Dulloo

**Affiliations:** 1 Pathology, Bundelkhand Medical College, Sagar, IND; 2 Anatomy, Gajra Raja Medical College, Gwalior, IND; 3 Pathology, Government Medical College Ratlam, Ratlam, IND; 4 Physiology, Parul Institute of Medical Sciences and Research, Parul University, Vadodara, IND

**Keywords:** reflective writing, kirkpatrick model, rubrics, workshop, portfolio

## Abstract

Purpose

The portfolio can be used as a crucial tool for self-reflection, which allows us not only to showcase achievements but also course correct on our personal and professional journey. However, there is a significant lack of awareness among medical professionals about portfolios. Arranging a workshop to impart this knowledge could be a potential mitigation approach. This study aims to assess the impact of workshops on portfolios on students' and faculty's knowledge. In addition, the study also analyzes the effect of using rubrics on reflective writing skills.

Method

A portfolio workshop was organized for the medical faculty and students in the Bundelkhand Government Medical College, Sagar, M.P. The Kirkpatrick model of training evaluation along with a rubric for the evaluation of reflective writing skills were used to measure the effectiveness of the workshop. Pre and post-tests for the workshop, pre and post-reflective writing skills, and workshop feedback were collected using questionnaires. The Shapiro-Wilk test and the Wilcoxon signed rank test were applied to the data collected.

Results

Out of 89 registrations for the workshop, only 81 people consented to the workshop and participated in the study. The total number of faculty was only 17 and the rest were students from all the phases. Both the Shapiro-Wilk test and the Wilcoxon signed rank test showed a significantly small p-value, stating that there was a significant positive impact on the knowledge, perception, and effectiveness of the workshop.

Conclusion

This study clearly outlines the positive impact of conducting a workshop on portfolios. A significant increase in participants' knowledge of portfolios is identified. Similarly, employing rubrics has a significant increase in the quality of reflective writing skills.

## Introduction

Effective professional development is crucial for the success of both medical students and faculty members. In the past, medical education heavily relied on lectures and standardized assessments as methods to evaluate students' comprehension and abilities. Many different approaches focused on improving advanced learning have been developed to equip aspiring healthcare professionals for future demands effectively. Among these, the use of portfolios is a crucial tool that not only assists healthcare professionals in their academic endeavors but also can enable them to maintain discipline and focus throughout their lives [1].

Traditionally, a portfolio serves as a means of introducing ourselves and showcasing our personality. It has been heavily used in the fields of design and business [2,3]. However, a medical portfolio comprises a collection of papers, materials, and proof that showcases the advancement, growth, milestones, and achievements in one's training and professional journey. A portfolio is efficacious in reflection, self-directed learning, and holistic skill enhancement, thus fostering creativity, effective content delivery, etc.

Portfolio-based learning is consistent with the ideas of andragogy, which was developed by Malcolm Knowles with adult learners in mind [2]. Additional attributes, such as attitudes, critical thinking, and ongoing professional development, can also impact learning; however, it is believed that promoting introspection and enabling further investigation will promote more successful learning [3,4].

Around the world, the majority of medical education institutes mandate that undergraduates complete a portfolio as part of their curriculum, and through several continuous training programs, instructors receive professional instruction to become excellent reflective writers. As per earlier research, portfolios help teachers evaluate students more holistically by allowing them to consider not just their academic performance but also their personal and professional growth [5,6].

As effective delivery of medical education is taking center stage in healthcare institutions, there is a growing focus on enabling medical professionals, both faculty and students, with the know-how of the right set of tools [4]. Workshops on portfolio development can not only invigorate the interest of faculty and students but also enable them to use this tool effectively.

However, this study is conducted to ascertain the effect of a workshop on portfolio toward participant comprehension. Two objectives of this study are: (1) To assess the impact of the workshop on portfolios and students' and faculty's knowledge; (2) To analyze the effect of using rubrics on reflective writing skills.

## Materials and methods

Overview

This quantitative evaluation study was conducted to assess pre and post-changes in participants’ knowledge/awareness of portfolios due to a workshop on medical portfolios. A four-hour long workshop was conducted at the Bundelkhand Government Medical College, Sagar, M.P. Subsequently, pre and post-changes in knowledge and feedback were collected and evaluated. The study was conducted after receiving due approval from the Institute's Ethical Committee. The approval number is IECBMC/DHR/2023/27, dated 20/10/2023.

Participants

All undergraduate and postgraduate medical students and faculty members were the potential population of this study. Participants were recruited by posting information about the workshop via notice boards and different WhatsApp groups. As this study is based on voluntary participation, a convenience sample of attendees (n = 81) was achieved.

Inclusion criteria

All medical students who were enrolled in graduate and post-graduate programs at the medical college and all the faculty members employed at the medical college were considered as potential participants. No prior experience with portfolio development workshops was a mandate. In addition, those who gave written consent, and completed pre- and post-assessments and feedback were included in the study.

Exclusion criteria

Medical students and faculty members who attended the workshop and did not provide written consent to participate in the study were excluded, as were participants who had prior experience of attending a workshop on medical portfolios or reflective writing.

Material

Presentations in the form of PowerPoints (Microsoft Corporation, Redmond, WA, US) were used to deliver the content of the workshop. Written informed consent was taken from the interested participants in paper format. Pre and post-evaluation and feedback from participants were collected using Google Forms. For the evaluation of reflective writing skills, a rubric was prepared for evaluation.

Procedure

**Figure 1:**
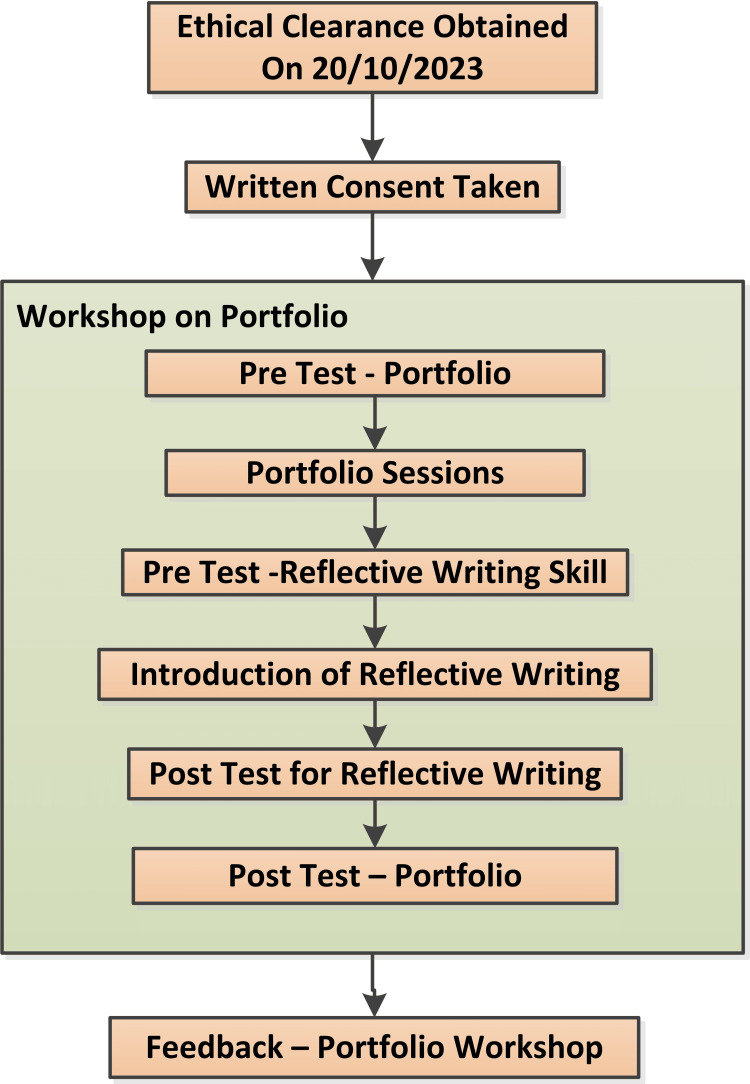
Schedule of events for the workshop on portfolios

Participants were informed about the workshop via notice boards and different WhatsApp groups. At the time of registration for the workshop, the participants were asked to sign written informed consent. The pre-existing knowledge of the participants about portfolios was assessed through a pre-test consisting of multiple-choice questions. Various key topics, such as the purpose of portfolios, the process of developing a model portfolio, reflective practice, clinical case writing, and the benefits of maintaining a medical portfolio, were delivered in the workshop. To evaluate the change in participant's ability to write reflections, before and after the reflective practice session, the participants were also asked to write reflectively about a life-changing incident in their lives. To assess the change in the awareness about portfolios due to knowledge imparted in the workshop, a post-test consisting of multiple choice questions was conducted. Finally, feedback on the workshop was collected from the participants. Figure 1 represents the schedule of the portfolio workshop.

The face validity of the questionnaires was performed by the medical education unit and curriculum committee members for relevance, clarity, and appropriateness.

Analysis

An analytical rubric was developed to assess the quantitative change in the reflective skills of the participants. Both pre and post-reflective writing scripts were evaluated using the rubric. Different weights were assigned to different criteria in the rubric. The highest weight was given to Comprehensive Coverage. It measured the extent of reflective details provided by the writer. Session facilitators performed all the evaluations. The rubric developed for this study is presented in Table 1.

**Table 1:** Analytical rubric to assess reflective writing skill The numbers under Criteria and Level of Information represent the corresponding weightage. To calculate the overall score in a given reflective writing criteria, based on assessment, first a “Level of Information” will be assigned and then the corresponding weightage will be multiplied by the weightage of those “Criteria”.

Criteria	Level of Information			
	Needs Improvement (1)	Developing (2)	Sufficient (3)	Above Average (4)
Comprehensive Coverage (x4)	Lack of reflection with no details.	Minimal reflection including a few supporting details and examples.	General reflection including some supporting details and examples.	In-depth reflection including supporting details and examples.
Clarity (x2.5)	The work appears undefined, with ideas not centered on supporting the composition, and thoughts seem disconnected.	The work's central purpose is clearly defined, and ideas are primarily focused to support the composition.	The work's central purpose is clearly defined, with ideas consistently focused on supporting the composition, with relevant details illustrating the author's ideas.	The work's central purpose is clearly defined, with well-focused supporting ideas and relevant details that enrich the overall content.
Organization (x2)	Writing is unclear and disorganized. Thoughts make little to no sense.	Writing is unclear, and thoughts are not well organized. Thoughts are not expressed logically.	Writing is mostly clear, concise, and organized with the use of excellent sentence/paragraph structure. Thoughts are expressed logically.	Writing is clear, concise, and well organized with the use of excellent sentence/paragraph structure. Thoughts are expressed logically.
Mechanics (x1.5)	There are numerous spelling or grammar errors per page of writing reflection.	There are more than five spelling or grammar errors per page of writing reflection.	There are no more than five spelling or grammar errors per page of writing reflection.	There are no more than three spelling or grammar errors per page of writing reflection.

## Results

The workshop was attended by 89 participants; however, only 81 gave consent for participation in the study. Forty-two of them were females and 39 were males. A total of 64 students and 17 faculty members participated. Details of the demographic distribution of the same are depicted in Table 2.

**Table 2:** Demographic distribution of participants

S No	Category	Sub-category	Gender	Sub-total	Total
1	Student	MBBS Student	Male	25	64
2			Female	32	
3	Student	Post-Graduate Student	Male	2	
4			Female	5	
5	Faculty	Assistant Professor	Male	7	17
6			Female	3	
7	Faculty	Associate Professor	Male	3	
8			Female	1	
9	Faculty	Professor	Male	2	
10			Female	1	
Total					81

The effectiveness of the workshop on Learning and Reflective writing is presented in Table 3. For Learning, effectiveness is established by significant improvement in pre and and post-test data. As the sample size (n=81) is significantly higher than 25, the general Z-score test is used instead of the Mann-Whitney test for ordinal data. A Z-score of 26.234 with a p-value ~0.0000 signified a significant increase in the mean of the post-test when compared to the pre-test. The p-values for pre and post-test data, using the Shapiro-Wilk test, are approximately ~0.0000. As p-values are very small (< 0.05) for both pre and post-test, it can be easily inferred that distribution is NOT normal distribution. Thus rejecting the NULL hypothesis. The statistical value of the Wilcoxon signed-rank test, 15.30, with a p-value of 1.25x10-12 indicates that almost for all the pre-test values, corresponding post-test values are lower, indicating significant improvement in effectiveness. The Cronbach alpha of the questionnaire comes out to be 0.732.

**Table 3:** Key statistics to assess the effectiveness of the workshop on portfolios Learning Level is the measure of knowledge about the portfolio of participants. Reflective Writing is the measure of the reflective writing skill of the participants.

Variable	Mean Distribution (Pre vs Post)		Distribution Check (Shapiro Test)				Wilcoxon Signed-Rank Test	
			Pre Data		Post-Data		Statistic	p-value
	Z-Score	p-Value	Statistic (W)	p-value	Statistic (W)	p-value		
Learning Level	26.234	~0.0000	0.895	~0.0000	0.809	~0.0000	15.30	~0.0000
Reflective Writing	25.060	~0.0000	0.977	~0.0000	0.943	~0.0000	12.06	~0.0000

For Reflective Writing, effectiveness is established by significant improvement in pre and post-test data. A Z-score of 25.060 with a p-value of ~0.0000 signified a significant increase in the mean of the post-test when compared to the pre-test. The p-values for pre and post-test data, using the Shapiro-Wilk test, is approximately ~0.0000. As p-values are very small (< 0.05) for both pre and post-test, it can be easily inferred that distribution is NOT normal distribution, thus rejecting the NULL hypothesis. The statistical value of the Wilcoxon signed-rank test is 13.92 with a p-value of approximately ~0.0000, indicating that for almost all the pre-test values, corresponding post-test values are lower, indicating significant improvement in effectiveness.

**Figure 2:**
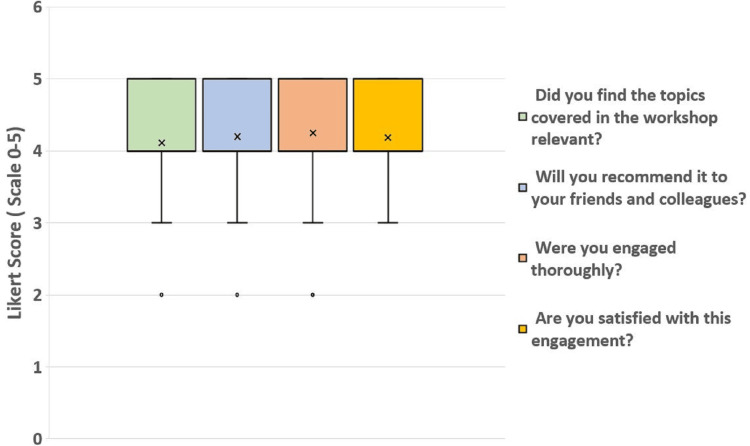
Workshop effectiveness based on feedback received from participants The score on the Y-axis represents scores provided by respondents on the Likert scale from 0-5; 0 means "Least" and 5 means "Most."

Data from the feedback reflects the effectiveness of the workshop on the Reaction Level of the checklist. Figure 2captures the distribution of responses to the relevant questions using a box and whiskers plot. The average collective score for the responses is 4.19 ± 0.80. About 79.3% of the responses were either 4 or 5.

The analytical Rubric mentioned in Table 1 is used for the evaluation of reflective writing scripts for both the pre and post-sessions. The distribution of reflective writing scores is presented inFigure 3. From the figure, a significant increase in the score for post-session is quite easily noticed. This effect is quite noticeable for the Comprehensive Coverage category.

**Figure 3:**
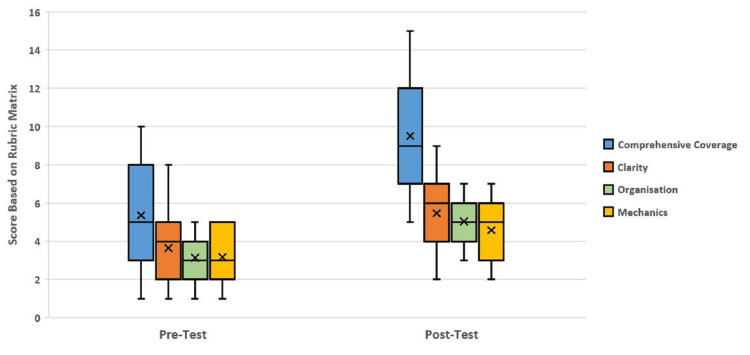
Distribution of the Reflective Writing Skills scores based on the rubric evaluation p-Values for all Criteria for Reflective Writing skill are ~ 0.0000

The quantitative change in the Learning Level of participants was assessed with the help of the Likert scale. Analysis of pre-test and post-test data of different MCQs reveals a significant increase in the mean values. At the same time, there is a noteworthy reduction in the standard deviation on responses. The p-values for all MCQs are very small (< ~0.0000) indicating a significant improvement in learning as a result of the workshop on portfolios. Detailed statistics on MCQs asked to assess the quantitative change in the Learning Levels of participants are covered in Table 4.

**Table 4:** Statistics on MCQs asked to assess the quantitative change in the Learning Levels of participants Participants responded to each MCQ using a Likert scale with 1= Least Likely and 5 = Most Likely.

S No	Question	Test	Mean	SD	p-Value
1	Is effectively communicating specialized skills and expertise is important for a medical portfolio?	Pre-Test	2.51	1.26	~0.0000
		Post-Test	4.14	0.8	
2	Do you agree that highlighting academic qualifications and training in a medical portfolio is crucial?	Pre-Test	2.7	1.3	~0.0000
		Post-Test	3.93	0.83	
3	Is including continuing education and professional development experiences in a portfolio important?	Pre-Test	2.58	1.25	~0.0000
		Post-Test	4.19	0.96	
4	Do you think that having a well-maintained portfolio would be beneficial for your future career or academic pursuits?	Pre-Test	2.8	1.36	~0.0000
		Post-Test	4.04	0.76	
5	Is a portfolio essential for showcasing clinical experiences in a medical field?	Pre-Test	2.6	1.24	~0.0000
		Post-Test	3.99	0.84	
6	Does a portfolio need to address ethical considerations and patient confidentiality?	Pre-Test	2.51	1.19	~0.0000
		Post-Test	4.06	0.81	
7	How confident are you that this workshop will enhance your understanding of portfolios?	Pre-Test	2.59	1.33	~0.0000
		Post-Test	4.09	0.85	
8	Should a portfolio be used as a tool to assess course and program learning outcomes?	Pre-Test	2.75	1.21	~0.0000
		Post-Test	3.95	0.74	
9	Will the portfolio modify course-learning outcomes?	Pre-Test	2.6	1.31	~0.0000
		Post-Test	3.93	0.86	
10	Should the portfolio be used as evidence for program accreditation processes?	Pre-Test	2.65	1.28	~0.0000
		Post-Test	4.05	0.8	

## Discussion

Various program evaluation models possess their unique strengths and weaknesses when it comes to assessing training activities. However, research has proven that Kirkpatrick's program evaluation model reigns supreme over other models [7,8]. For this reason, we turned to Kirkpatrick's model to thoroughly evaluate the healthcare staff's overall response to our workshop on portfolios, as well as its impact on their learning and behavior.

According to the workshop evaluation, participants were quite pleased with the workshop (Level 1). A study by Rabiee et al. found that half of the staff at the Arak University of Medical Sciences believed the workshop was excellent while the other half rated it as moderate or weak [9]. Further research by Ofoghi et al. using Kirkpatrick's model also showed that the physical conditions of the classroom had a direct and positive impact on the quality of learning, as reported by librarians after a short-term training course [10].

This study has incorporated the first two levels of Kirkpatrick’s cycle, namely, Reaction and Learning. This decision was taken due to a time shortage. Similar findings were present in Faysal et al.'s study [11]. Their study covered the effect of portfolio workshops up to the first two levels of Kirkpatrick’s cycle. However, the difference between the participants of the two studies is that they conducted a faculty development program (FDP) and our study involved both students and faculty alike. In addition, they have also used the Gibbs reflective cycle for the evaluation of reflective writing skills and our study employed an analytical rubric.

In a study reported by Belay et al. in 2021, a workshop was organized very similar to that of ours [12]. It was divided into PowerPoint presentations, along with hands-on activity coupled with pre-test, feedback, and post-test. A similar layout of the workshop has been utilized by many other studies [13-15]. In contrast to our study, the primary objective of these studies was to utilize portfolios to evaluate learning objectives and the growth of abilities and competencies by utilizing frequent feedback and student reflection.

Similar to our study, Cheng et al. (2019), Kondo et al. (2022), and Andrade (2000) also employed rubrics to analyze reflective and analytical performance. It is found to have a significant positive impact on the academic performance of students [16-18].

Limitations

Only the first two of the four levels outlined by the Kirkpatrick model of training evaluation are evaluated in this study. Thus, a study over a long duration will help in precisely measuring the effects of portfolio workshops on medical students and faculty at two more levels, namely, Behaviors and Results.

In addition, the validity of the questionnaire, used for pre-test, post-test, and feedback, should be established not just by face validity but also by construct, content, and criterion validity. Also, the portfolios are time-consuming for both the assessor and the individual being assessed and they are difficult to mark.

With more time, a questionnaire with even stronger reliability can be created.

## Conclusions

This study clearly outlines the positive impact on faculty and students of conducting a workshop on portfolios. Statistical evaluation of the participant's responses delineates a significant increase in participants' knowledge of portfolios. Similarly, employing a rubrics matrix for evaluation significantly increases the quality of reflective writing skills.
